# Early childcare from 0 to 3 years and child behavioural difficulties at age 5.5 years in France, data from the ELFE mother-child cohort

**DOI:** 10.1192/j.eurpsy.2023.1498

**Published:** 2023-07-19

**Authors:** A. R. Gomajee, K. M. Barry, M. Melchior

**Affiliations:** 1 Social Epidemiology Research Team, Sorbonne University, INSERM U1136, IPLESP, Paris; 2 EHESP, Rennes, France

## Abstract

**Introduction:**

Previous studies have showed that the type of early childcare can be associated with child behavioural difficulties though results vary across countries.

**Objectives:**

To investigate the link between early childcare from birth to 3 years and child behavioural difficulties at age 5.5 years, in the French context.

**Methods:**

In this study (n = 9,699), parents participating in the French ELFE birth cohort reported their child main childcare type used between birth and three years of age (centre-based (22.6%), childminder (43.6%), informal (8.2%) or parents only [25.7%)), and the child’s behaviour through the Strengths and Difficulties Questionnaire (SDQ) at age 5.5 years. Scores were calculated for each SDQ subscale as well as the total SDQ scores. Logistic regression analyses were carried out adjusting on socio-demographic, parents’ and child’s characteristics to evaluate the association between early childcare type and abnormal SDQ total score (>16) as well as subscale scores.

**Results:**

In the study population, 584 (6.02%) children had abnormal SDQ total score, and 1,104 (11.4%) in the emotional subscale, 573 (5.91%) in the peer relationship subscale, 1,433 (14.8%) in behavioural subscale, and 1,097 (11,3%) in the hyperactivity subscale. After adjusting, compared to children who were looked after by their parents only, those who were in centre-based childcare had a lower likelihood of having an abnormal SDQ total score (OR_a_ = 0.76 [95% CI: 0.58 – 0.99]), while there was no significant difference for children who were in a childminder’s care (OR_a_ = 0.94 [95% CI: 0.75 – 1.17]) or in an informal childcare (OR_a_ = 1.18 [95% CI: 0.86 – 1.63]). In additional analyses, we found that compared to children in parental care only, children in centre-based childcare had a decreased likelihood of having abnormal internalising subscales scores: emotional subscale, (OR_a_ = 0.81 [95% CI: 0.67 – 0.99]) and peer relationship subscale, (OR_a_ = 0.79 [95% CI: 0.61 – 1.02]). All other associations were not significant except for the informal childcare which was associated to a higher likelihood of abnormal behavioural subscale (OR_a_ = 1.29 [95% CI: 1.03 – 1.62]).

**Conclusions:**

In the French ELFE cohort, early centre-based childcare was linked to lower likelihood of having internalising problems in children at age 5.5 years. Further studies should focus on the possible mecanisms of this association. Family and childhood policies should aim to make centre-based childcare accessible to more children.

**Disclosure of Interest:**

None Declared

